# Comprehensive knowledge of mother-to-child HIV/AIDS transmission, prevention, and associated factors among reproductive-age women in East Africa: insights from recent demographic and national health surveys

**DOI:** 10.1186/s12905-024-03173-1

**Published:** 2024-06-01

**Authors:** Bewuketu Terefe, Mahlet Moges Jembere, Bikis Liyew

**Affiliations:** 1 Department of Community Health Nursing, School of Nursing, College of Medicine and Health Sciences, University of Gondar, Gondar, Ethiopia; 2 Department of Emergency and Critical Care Nursing, School of Nursing, College of Medicine and Health Sciences, University of Gondar, Gondar, Ethiopia

**Keywords:** Comprehensive knowledge, Mother-to-child transmission, HIV/AIDS prevention, Reproductive-age women, East Africa, Associated factors

## Abstract

**Background:**

More than 90% of babies acquire HIV/AIDS through vertical transmission, primarily due to low maternal comprehensive knowledge about Mother-To-Child Transmission (MTCT) of HIV/AIDS and its prevention, which is a cornerstone for eliminating MTCT of HIV/AIDS. However, there are limitations in terms of population data and literature evidence based on recent Demographic and Health Surveys (DHS) reports in East Africa. Therefore, this study aims to assess the comprehensive knowledge and PMTCT of HIV/AIDS among women, as well as the associated factors in East Africa.

**Methods:**

Our data was obtained from the most recent DHS conducted in East African countries between 2011 and 2022. For our research, we included DHS data from ten nations, resulting in a total weighted sample of 133,724 women for our investigation. A generalized linear model (GLM) with a log link and binomial family to directly estimate prevalence ratios (PR) and 95% confidence intervals (CI) for the association between the independent variables, and the outcome variable. Finally, we reported the adjusted prevalence ratios along with their corresponding 95% CIs. Factors with p-values ≤ 0.2 for univariate logistic regression and < 0.05 were considered statistically significant factors of HIV/AIDS knowledge and prevention in the final model.

**Results:**

In this study, 59.41% (95% CI: 59.15–59.67) of respondents had a comprehensive knowledge about MTCT of HIV/AIDS and its prevention among reproductive-age women in East Africa. Being in the older age group, better education level, being from a rich household, employment status, having ANC follow up, institutional delivery, and modern contraception usage were associated with higher prevalence ratios of comprehensive knowledge about MTCT of HIV/AIDS and its prevention. However, being single in marital status, rural women, and traditional contraception utilization were associated with lower ratios of comprehensive knowledge about MTCT of HIV/AIDS and its prevention.

**Conclusion:**

Our findings indicate a significant deficiency in comprehensive knowledge and prevention of HIV/AIDS MTCT among women in East Africa. These results emphasize the need for significant improvements in maternal-related health services. It is crucial to effectively target high-risk populations during interventions, raise awareness about this critical public health issue, and address the catastrophic consequences associated with MTCT. By implementing these measures, we can make substantial progress in reducing the transmission of HIV/AIDS from mother to child and ensuring better health outcomes for both mothers and their children.

## Introduction

Vertical transmission of Human Immunodeficiency Virus (HIV) from mother to child during pregnancy, birth, and breast feeding remains a serious public health concern and is the leading source of HIV infection in children under the age of 15 worldwide [1, 2]. Morbidity and mortality from HIV infection have declined globally over the last decade as a result of preventive measures such as greater coverage of Antiretroviral Therapy (ART) and prevention of HIV/AIDS transmission from mother to child (PMTCT) [3, 4]. However, over 90% of new infections of HIV in babies and young children are transmitted from mother to child still [5]. In 2022, there was around 39 million HIV-positive people worldwide [6]. Among these, about 37.5 million, and 1.5 million were adults and children (15 and under), 53% were women and girls [6, 7]. Similarly, the USAIDS, in 2023 estimated, more than 39 million individuals were infected with HIV, and lived with the virus [8]. Additionally, AIDS-related illnesses claimed the lives of almost 630 thousand people this year [8]. However, Eastern and Southern Africa making over half of that number [9, 10]. Using the above references, it is obvious that the number of people getting infected with HIV is increasing over time, and rigorous research related to it is expected from various individuals and organizations [6].

According to the UNAIDS 2023 report, in terms of women and girls in 2022, women and girls of all ages accounted for 46% of all new HIV infections worldwide [11]. Women and girls (of all ages) accounted for 63% of all new HIV infections in Sub-Saharan Africa (SSA) [6, 11]. In all other geographical regions, men and boys accounted for more than 70% of new HIV infections in 2022. In 2022, 4000 adolescent girls and young women aged 15–24 years would be infected with HIV per week over the world. SSA was responsible for 3100 of these illnesses [12].

In 2017, approximately 50% of the 180,000 new pediatric HIV infections occurred during breastfeeding, and it is estimated that in the absence of any intervention to prevent MTCT, the risk of transmission ranges from 15 to 45% (5–10% during pregnancy, 10–20% during childbirth, and 10–20% via mixed infant feeding) [13]. This rate, however, can be reduced to less than 5% with appropriate interventions [13]. The recent 2023 UNAIDS reports indicated that, each day, HIV infection affects 4,000 individuals, including 1,100 young people aged 15 to 24. If present patterns persist, it is projected that 1.2 million individuals will acquire HIV in 2025, which is three times higher than the targeted number of 370,000 new infections for that year [14].

Knowledge of MTCT and PMTCT for HIV/AIDS is associated with characteristics such as maternal age, maternal education, wealth level, occupation, marital status, media exposure, and domicile [15–20]. Maternal awareness of HIV/AIDS MTCT and prevention is essential for HIV MTCT elimination. Despite the fact that the majority of the population in SSA lives in rural areas with limited availability and accessibility of health facilities, the majority of studies on HIV/AIDS knowledge and prevention were conducted among available women, such as those who came to the health facility for their antenatal care follow up [17, 21–25]. Since East Africa is the second most affected region by HIV/AIDS, women are the primary vulnerable group among the population in the region, and no current study has revealed the situation utilizing nationally representative data from recent DHS surveys that this study aims to investigate. Hence, studying women’s comprehensive knowledge about HIV/AIDS will help reduce stigma and discrimination, improve health outcomes for mothers and children, and decrease MTCT [26]. Furthermore, by understanding the factors involved, the findings of this study can provide valuable insights for policymakers, healthcare providers, and public health practitioners in East Africa. Therefore, using the recent national demographic health survey data, this study aimed to assess the comprehensive knowledge and PMTCT of HIV/AIDS among women, as well as its associated factors in East Africa.

## Methods

### Data sources and study population

Our data was obtained from the most recent Demographic and Health Surveys (DHS) conducted in East African countries between 2011 and 2022. This study included DHS data from 10 countries as shown in Table 1. To conduct our research, we incorporated DHS data from these 10 nations using the corresponding Stata command. The survey utilized stratified, two-stage cluster sampling. In the first step, enumeration areas (EAs) were selected with a probability proportional to their size within each sampling stratum. Subsequently, households were sampled in the second step. The source population consisted of mothers of reproductive age. Consequently, classical logistic regression was deemed more appropriate. Ultimately, our study utilized a weighted sample of 133,724 women of reproductive age.

**Table 1:** Countries, sample size, and survey year of Demographic and Health Surveys included in the analysis for ten East African countries

Country	Survey year	Sample size(weighted)	Frequency(weighted)
Burundi	2016/17	16,128	12.06
Ethiopia	2016	12,673	9.48
Comoros	2012	3,714.205	2.78
Madagascar	2021	12,881	9.63
Malawi	2016/17	22,239	16.63
Mozambique	2011	11,486	8.59
Rwanda	2019/20	14,386	10.76
Uganda	2016	17,929	13.41
Zambia	2018	12,688	9.49
Zimbabwe	2015	9,600	7.18

### Data management and statistical analysis

Stata version 17 is used to extract, recode, and analyze data. Weighting was used throughout the study to ensure representativeness and non-response rate, as well as to obtain a suitable statistical estimate (robust standard error) [27]. In the univariate analysis, variables with a p-value of ≤ 0.2 were considered for the multivariable analysis. The multivariable logistic model provided the adjusted prevalence ratio (APR) with a 95% confidence interval to identify the associated factors of knowledge of PMTCT use. We used generalized linear models (GLM) with a log link and binomial family to directly estimate prevalence ratios (PR) and 95% confidence intervals (CI) for the association between the independent variables and the binary outcome of comprehensive knowledge of PMTCT. This approach allows for the estimation of PRs without the common issue of overestimation that can occur when using logistic regression to estimate odds ratios for common outcomes. We specified robust standard errors to account for potential heteroscedasticity in the model. The log-binomial GLM allowed us to directly estimate prevalence ratios, which are more readily interpretable than odds ratios for this cross-sectional study with a relatively common outcome. The use of robust standard errors ensures valid statistical inferences in the presence of any violation of model assumptions.

Since the data had a potential hierarchical structure, we assessed it to determine if multilevel model analysis could be conducted by calculating the intra-class correlation (ICC) coefficient. However, the ICC coefficient was found to be only approximately 1.7%, which did not meet the minimum criterion for conducting multilevel analysis. Descriptive data were summarized using measures such as frequency count and proportion for categorical variables. To examine multicollinearity among the independent variables, a logistic regression was fitted using the variance inflation factor. The Hosmer and Lemeshow test were also used to evaluate the overall fitness of the final regression model. The statistical significance for the final model was set at *p* < 0.05.

### Variables of the study

#### The outcome variable

The outcome variable of this study was the comprehensive knowledge of PMTCT among women of reproductive age. This outcome was measured using two percentages: the percentage of women who were aware that HIV can be transmitted from mother to child during pregnancy, delivery, and breastfeeding, and in all three ways; and the percentage of women who knew that the risk of mother-to-child transmission can be reduced by the mother taking special drugs. Women who responded “Yes” to both questions were considered knowledgeable about PMTCT, whereas those who missed either of them were classified as not knowledgeable. The study population included all women of reproductive age, specifically those aged 15–49 years old, as determined by the IR file, and the time period was defined by the current status at the time of the survey interview. The outcome variable was subsequently recategorized as “Yes = 1” if the women knew the correct answers to both questions, and “No = 0” if they missed either of them. All classifications and analyses were conducted following the guidelines provided in the DHS statistics book [28].

### The independent variables

Independent variables: Various maternal-related factors were included, such as maternal age, educational status, types of places of residence, marital status, household wealth index, current employment status, mass media exposure, ANC follow-up, place of delivery, number of health visits in the past 12 months, under-five children, contraceptive utilization, distance to the health facility, knowledge of HIV/AIDS, sex of the household head, country, and breastfeeding status.

## Results

### Sociodemographic characteristics of the study participant

In this study, a total weighted sample of 133,724 women of reproductive age were enrolled in East African countries. Nearly half of them, 53,712 (40.17%), fell within the 15–24 years age group. In terms of marital status, approximately half of the mothers, 66,037 (49.38%), were married. Regarding place of residence, educational status, wealth index, place of delivery, and ANC follow-up, the majority of mothers, 97,636 (73.01%), 97,637 (46.81%), 34,309 (25.66%), 120,494 (90.11%), and 129,855 (97.11%), respectively, were from rural areas, had primary educational status, belonged to the richest households, opted for institutional delivery, and had at least one ANC follow-up during their pregnancies. Similarly, approximately 67,551 (50.52%) and 79,879 (59.73%) of women did not have access to any form of mass media exposure (such as radio, television, or magazines/newspapers) and were unemployed, respectively. However, more than half of the mothers, 88,376 (66.09%), and 51,509 (38.52%), did not utilize any contraceptive methods and reported facing challenges related to the distance to the health facility. Furthermore, around 107,992 (80.76%) participants had only one health facility visit per year, and 93,094 (69.62%) reported having male household heads (Table 2).

**Table 2:** Socio-demographic and maternal related characteristics of respondent’s contraception methods utilization among married women in East African countries (weighted *n* = 133,724)

Variables	Categories	Frequency	Percentage
Age in years	15–24	53,712	40.17
	25–34	42,216	31.57
	35–49	37,796	28.26
Residence	Urban	36,088	26.99
	Rural	97,636	73.01
Mothers’ Educational status	No education	23,375	17.48
	Primary	62,593	46.81
	Secondary and higher	47,756	35.61
Mothers’ occupation	Not working	53,845	40.27
	Working	79,879	59.73
Wealth index	Poorest	22,204	16.60
	Poorer	23,899	17.87
	Middle	25,259	18.89
	Richer	28,053	20.98
	Richest	34,309	25.66
Mass media exposure	No	67,551	50.52
	Yes	66,173	49.48
Number of under five children	No	43,759	32.72
	1–2	80,984	60.56
	More than two	8,981	6.72
Contraceptive methods	No method	88,376	66.09
	Traditional methods	3,255	2.43
	Modern methods	42,094	31.48
Number of health visits in the past 12 months	Once	107,992	80.76
	More than one	25,732	19.24
Distance from health facility	A big problem	51,509	38.52
	Not a big problem	82,182	61.46
Currently breastfeeding	No	98,254	73.48
	Yes	35,470	26.52
Marital status	Never married	35,719	26.71
	Married	66,037	49.38
	Divorced/widowed	31,968	23.91
Place of delivery	Home	13,230	9.89
	Health facility	120,494	90.11
ANC follow up	No	3,869	2.89
	Yes	129,855	97.11
Sex of the household head	Male	93,094	69.62
	Female	40,630	30.38

### Knowledge of women about PMTCT of HIV/AIDS

The overall comprehensive knowledge of PMTCT of HIV/AIDS was about 79,447(59.41%). The transmission of HIV/AIDS during pregnancy 110,349(82.52%), during delivery 120,735(90.29%), during breastfeeding 119,955(89.70%), and about a special drug to avoid HIV during pregnancy 108,782(81.35%) was replied correctly (Table 3).

**Table 3:** Knowledge about the prevention of mother to child transmission of HIV/AIDS and its prevention among reproductive-age women in East Africa

Knowledge of MTCT and PMTCT	Categories	Number of respondents (*N* = 133,724)	Percentage
HIV transmitted during pregnancy	No	23,375	17.48
	Yes	110,349	82.52
HIV transmitted during delivery	No	12,989	9.71
	Yes	120,735	90.29
HIV transmitted during breastfeeding	No	13,769	10.30
	Yes	119,955	89.70
There are special drugs to avoid HIV transmission to the child during pregnancy (PMTCT)	No	24,942	18.65
	Yes	108,782	81.35
Comprehensive knowledge of MTCT and PMTCT	Not knowledgeable	54,277	40.59
	Knowledgeable	79,447	59.41

### Factors associated with comprehensive knowledge of PMTCT of HIV/AIDS among women in East Africa

The adjusted prevalence ratio (APR) of having comprehensive knowledge about PMTCT of HIV increased by 1.09 times (APR = 1.09, 95% CI: 1.07, 1.11) and 1.05 times (APR = 1.05, 95% CI: 1.03, 1.08) among women aged 25–34 years and 35–49 years, respectively, compared to women aged 15–24 years. Similarly, compared to participants with no education, mothers who had completed primary education and secondary/higher education had higher prevalence ratios of being knowledgeable about PMTCT of HIV, with prevalence ratios of 1.08 (APR = 1.08, 95% CI: 1.05, 1.10) and 1.06 (APR = 1.06, 95% CI: 1.03, 1.13) respectively. Regarding the household wealth index, mothers from middle, richer, and richest households showed higher ratios of having comprehensive knowledge of PMTCT of HIV compared to mothers from the poorest households, with prevalence ratios of 1.06 (APR = 1.06, 95% CI: 1.02, 1.11), (APR = 1.09, 95% CI: 1.04, 1.13), and (APR = 1.08, 95% CI: 1.05, 1.11) respectively. The prevalence ratio of comprehensive knowledge about HIV were 1.04 times higher among employed mothers (APR = 1.04, 95% CI: 1.03, 1.06) compared to unemployed mothers. The ratios of knowledge about HIV among married and divorced/widowed women were (APR = 1.19, 95% CI: 1.15, 1.26) and (APR = 1.16, 95% CI: 1.14, 1.19) times higher, respectively, when compared to never married women. Women who gave birth at health institutions had 1.25 times higher ratios of (APR = 1.25, 95% CI: 1.23, 1.28) of being knowledgeable about PMTCT of HIV compared to those who gave birth at home. Moreover, women who had at least one ANC visit showed more comprehensive knowledge about PMTCT, with a prevalence ratio of 1.22 (95% CI: 1.17, 1.27) compared to those who did not have an ANC visit. On the other hand, regarding contraceptive method types, mothers who utilized traditional methods had 0.13 times lower ratios (APR = 0.87, 95% CI: 0.84, 0.91), while those who used modern methods had 1.09 times higher ratios (APR = 1.09, 95% CI: 1.07, 1.10), of being knowledgeable about PMTCT of HIV compared to mothers who did not use any type of contraceptives. Finally, women from rural areas showed less comprehensive knowledge about PMTCT, with a prevalence ratio of 0.98 (95% CI: 0.97, 0.99) compared to urban residential women (Table 4).

**Table 4:** Multivariable logistic regression analysis results on determinants of knowledge of PMTCT, and HIV/AIDS among reproductive age women in East Africa

Knowledge on PMTCT and HIV	No, *n* (%)	Yes, *n* (%)	Prevalence ratio	*P* > z	95%	CI
Explanatory Variables					Lower	Upper
Maternal age						
15–24	24,182(45.02)	29,530(54.98)	1			
25–34	15,561(36.86)		1.09	0.0001	**1.07**	**1.11**
35–49	14,535(38.46)	23,261(61.54)	1.05	0.0001	**1.03**	**1.08**
Maternal education						
Not educated	10,043(42.97)	13,332(57.03)	1			
Primary	24,327(38.87)	38,266(61.13)	1.08	0.0001	**1.05**	**1.10**
Secondary higher	19,907(41.68)	27,841(58.32)	1.06	0.007	**1.03**	**1.13**
Wealth index						
Poorest	9,172(41.30)	13,033(58.70)	1			
Poorer	9,909(41.46)	13,990(58.54)	1.03	0.144	0.99	1.07
Middle	10,295(40.76)	14,963(59.24)	1.06	0.0001	**1.02**	**1.11**
Richer	11,279(40.21)	16,774(59.79)	1.09	0.0001	**1.04**	**1.13**
Richest	13,622(39.70)	20,687(60.30)	1.08	0.0001	**1.05**	**1.11**
Mother employed						
No	23,656(43.93)	30,188(56.07)	1			
Yes	30,621(38.33)	49,259(61.67)	1.04	0.0001	**1.03**	**1.06**
Mass media exposure						
No	27,850(41.23)	39,700(58.77)	1			
Yes	26,426(39.94)	39,747(60.06)	0.99	0.054	0.98	1.01
Marital status						
Never married	17,018(47.64)	18,701(52.36)	1			
Married	25,895(39.21)	40,142(60.79)	1.19	0.0001	**1.15**	**1.26**
Divorced/widowed	11,364(35.55)	20,605(64.45)	1.16	0.0001	**1.14**	**1.19**
Place of delivery						
Home	6,739(50.94)	6,491(49.06)	1			
Health facility	47,537(39.45)	72,957(60.55)	1.25	0.0001	**1.23**	**1.28**
ANC follow-ups						
No	2,174(56.09)	1,695(43.81)	1			
Yes	52,103(40.12)	77,752(59.88)	1.22	0.0001	**1.17**	**1.27**
Number of health visits						
Once	43,955(40.70)	64,037(59.30)	1			
More than one	10,322(40.11)	15,410(59.89)	1.01	0.143	0.99	1.02
Contraceptive method types						
No method	37,980(42.98)	50,396(57.02)	1			
Traditional methods	1,513(46.49)	1,742(53.51)	0.87	0.0001	**0.84**	**0.91**
Modern methods	14,784(35.12)	27,310(64.88)	1.09	0.0001	**1.07**	**1.10**
Residence type						
Urban	14,087(39.03)	22,001(60.97)	1			
Rural	40,190(41.16)	57,446(58.84)	0.98	**0.009**	**0.97**	**0.99**
Distance to health facility						
A big problem	21,261(41.28)	30,248(58.72)	1			
Not a big problem	33,016(40.16)	49,199(59.84)	1.01	0.689	0.99	1.02

Where bolded confidence intervals indicates significant variables at *P* < 0.05 in the final model

## Discussion

The purpose of this study was to examine comprehensive knowledge regarding HIV/AIDS transmission from mother to child, as well as its prevention and associated factors, among reproductive-age women in East Africa using recent DHS data. In this survey, about 59.41% of respondents were comprehensively knowledgeable with HIV/AIDS MTCT and its prevention. This result is lower than in previous studies conducted in Zimbabwe [16], Tanzania [29], and Nigeria [30]. However, our study’s findings are slightly higher than those of research conducted in SSA [19], Ethiopia [17], and Uganda [31]. Firstly, the disparity may be due to the fact that the study conducted a pooled analysis that included data from multiple East African countries. Since each country may have different contexts, healthcare systems, and population characteristics, the combined analysis might have introduced variations in the results. Secondly, differences in the study time, sample size, outcome ascertainment criteria, approach of analysis, and the study population could contribute to the observed disparity. These methodological variations can influence the findings and interpretations. For example, if the studies were conducted at different time points, there could have been changes in healthcare policies, interventions, or awareness campaigns that could impact the knowledge levels about the specific topic being studied. Additionally, differences in sample sizes, criteria for determining the outcome, analytical approaches, and characteristics of the study population (e.g., age groups, socioeconomic status) can all introduce variations in the results. Overall, the observed disparity in the findings may be due to a combination of factors related to the diverse nature of the pooled analysis, as well as differences in study methodology and population characteristics. These factors need to be considered when interpreting and comparing the results of studies conducted in different settings or at different time points. In the multiple logistic regression analysis, older age, attendance at primary and secondary school, coming from a wealthy family, marital status, at least one ANC follow-up, institutional delivery, and contraception use were associated with a higher likelihood of knowing about HIV/AIDS MTCT and prevention.

The study found that older age groups had higher ratios of knowing about MTCT of HIV/AIDS and its prophylaxis than younger age groups (women aged 15–24 years). This is consistent with research conducted in SSA, Ethiopia, and Zimbabwe [16, 19, 20]. This could be linked to older women’s proximity to various maternal health services during each consecutive pregnancy. Furthermore, this could imply that initiatives to support younger women (adolescents) in raising HIV awareness, reducing MTCT, and promoting ART adherence and viral suppression are insufficient [13]. As a result, more attention should be placed on HIV/AIDS and MTCT ideas for those young moms in order to prevent HIV transmission from mother to child. The study’s findings regarding the association between age groups and knowledge about MTCT of HIV/AIDS align with the Social Cognitive Theory (SCT) proposed by Bandura (1986) [32]. According to SCT, individuals acquire knowledge and behavior through observational learning and social interactions. In this context, older women’s higher ratios of knowing about MTCT and its prophylaxis could be attributed to their increased exposure to maternal health services, which provide opportunities for information exchange and learning from healthcare professionals. This finding supports the notion that access to healthcare services and exposure to educational interventions play a crucial role in knowledge acquisition and behavior change. Furthermore, the paragraph suggests that the lack of sufficient initiatives targeting younger women, particularly adolescents, raises questions about the effectiveness of current interventions based on the Theory of Planned Behavior (TPB). According to TPB, individuals’ attitudes, subjective norms, and perceived behavioral control influence their intentions and subsequent behaviors [33].

Similarly, when compared to uneducated participants, women with primary and secondary/higher educational attainment had significantly higher likelihood of being knowledgeable about HIV PMTCT. This is consistent with prior research done elsewhere SSA [19], and Ethiopia [15, 20, 34]. This could be because educated women have better access to health-related information and can grasp HIV/AIDS and associated MTCT. The findings regarding the association between educational attainment and knowledge about HIV PMTCT align with several theoretical perspectives. One such framework is the Health Belief Model (HBM), which suggests that individuals’ health-related beliefs and perceptions influence their adoption of preventive behaviors. In this context, educated women may have a higher level of perceived susceptibility to HIV/AIDS and recognize the significance of PMTCT knowledge in protecting their own health and that of their children [35–37]. Education can also enhance their perceived benefits of adopting preventive measures, such as adhering to antiretroviral therapy and practicing safe delivery methods, leading to a higher likelihood of being knowledgeable about PMTCT [35]. Furthermore, the findings resonate with the Diffusion of Innovations theory, which posits that knowledge and new ideas are more readily adopted by individuals with higher education levels [36, 38].

In terms of the household wealth index, and employment status, the current study discovered that mothers from the middle, richer, and richest households were more likely to have comprehensive knowledge of HIV PMTCT than mothers from the worst household wealth index, and unemployed mothers respectively. This is consistent with research undertaken in SSA [19], Ethiopia [15], and Tanzania [39]. The higher degree of awareness among women from well-off households could be attributed to their easy access to maternal health services such as PMTCT programs and mass media exposure. Employed mothers may have more social interaction and independence than unemployed mothers.

In terms of marital status, married and divorced/widowed women were more educated about HIV PMTCT than never married women. Women who were married or divorced were more likely to have comprehensive understanding about MTCT and its eradication. This conclusion is similar with findings from Rwanda [40], Nigeria [41, 42], and Ethiopia [15, 43]. The most obvious explanation is that married and divorced women obtain health information at health care centers during ANC visits and related family planning services [15]. Women who gave birth in health facilities, those who used modern contraception, and those who had ANC follow-up during their pregnancy periods had a higher likelihood of understanding HIV PMTCT than their counterparts. This could be because women who have a history of ANC follow-up may have the opportunity to learn from health experts, and this information may improve women’s knowledge of PMTCT. Similarly, women with a history of institutional delivery and contemporary contraception use may be eligible for PMTCT services from health experts at a health facility. This finding is similar to the findings of an Ethiopian investigation [18, 44].

Women from rural areas in developing countries and Sub-Saharan Africa tend to exhibit lower comprehensive knowledge about Prevention of Mother-to-Child Transmission (PMTCT) of HIV compared to urban residential women. Research indicates that various factors influence this disparity in PMTCT knowledge among women in different settings. Studies have shown that women with access to mass media, formal education, and occupation are more likely to have correct knowledge of MTCT and PMTCT [15, 45]. Urban areas often provide better access to health information and education through media and workplaces, contributing to higher knowledge levels among urban women. Women’s decision-making power, wealth index, and occupation type play a significant role in their PMTCT knowledge [46, 47]. Women with decision-making power, manual occupations, and higher wealth status are more likely to have better PMTCT knowledge.

Factors like ANC follow-up and utilization of maternal health services are associated with higher PMTCT knowledge among women [45, 48]. Women who engage in ANC services have increased opportunities to learn about PMTCT from health professionals. Rural residents face challenges in accessing PMTCT services due to limited infrastructure and media coverage, contributing to lower knowledge levels compared to urban areas [45, 48]. Efforts are needed to intensify health education and PMTCT services in rural and emerging regions.

This study relied on nationally representative data, as well as adequate statistical analysis and a large number of factors. As a result, it can assist policymakers, as well as governmental and non-governmental groups, in making appropriate actions. However, the study had certain shortcomings. First, because it was based on survey data, some characteristics that may be related with the outcome variable, such as the quality and availability of health care and knowledge about HIV/AIDS, were not addressed. Second, because it is based on survey data, we are unable to demonstrate the temporal relationship between the result variable and the independent variables that were included. Furthermore, we used DHS from the preceding ten years, and there may have been changes in MTCT and ART regimen awareness, as well as ART uptake before to and during pregnancy (Option B+) over time. As a result, due to time constraints, caution is advised when interpreting study findings.

## Conclusions, and implications

The study findings reveal that HIV/AIDS MTCT and preventive knowledge among reproductive-age women in East Africa is rated as low. However, certain factors were identified to be associated with a higher likelihood of knowledge about MTCT of HIV/AIDS and its prevention. These factors include older age, attending primary and secondary school, coming from a wealthy family and rural areas, being married, having at least one antenatal care (ANC) follow-up, opting for institutional delivery, and using contraception.

These findings have important implications for addressing the knowledge gap and improving the prevention of HIV/AIDS MTCT among reproductive-age women in East Africa. The study highlights the need for targeted interventions and educational programs that focus on improving knowledge and awareness of HIV/AIDS transmission and prevention methods. Specifically, efforts should be directed towards younger women, those with limited education, and those from lower socioeconomic backgrounds, as they are more likely to have lower levels of knowledge.

Furthermore, the study underscores the importance of ANC utilization and institutional delivery, as these factors were associated with higher knowledge levels. Strengthening and expanding ANC services, particularly in terms of HIV/AIDS education and counseling, can enhance women’s understanding of MTCT and its prevention. Similarly, promoting contraception use among reproductive-age women can serve as an additional avenue to disseminate information on MTCT prevention.

Policy makers, healthcare providers, and public health practitioners in East Africa should consider incorporating these findings into their strategies and interventions. By addressing the identified factors and tailoring interventions to the specific needs of different subgroups, it is possible to improve knowledge levels, reduce stigma and discrimination, enhance health outcomes for mothers and children, and ultimately reduce the incidence of HIV/AIDS MTCT in the region. As a result, it is preferable to prioritize high-risk populations during the intervention in order to raise awareness about this critical public health issue and address its catastrophic consequences. Improving maternal-related services such as ANC, institutional delivery, and family planning are examples of good possibilities for women to have a more thorough understanding of HIV/AIDS vertical transmission.

## Data Availability

All data concerning this study are accommodated and presented in this document. The detailed data set can be freely accessible from the www.dhsprogram.com website.
